# *Lactiplantibacillus plantarum*-Fermented Broccoli Attenuates Metabolic Dysfunction and Improves Skeletal Muscle Quality in High-Fat/High-Fructose Diet-Fed Mice

**DOI:** 10.1021/acs.jafc.6c05322

**Published:** 2026-09-28

**Authors:** Ya-Ru Kuo, Tzu-Ling Lin, Pin-Yu Ho, Wei-Jen Chen, Hui-Fang Chu, Ming-Chung Tseng, Yu-Shan Wei, Min-Hsiung Pan

**Affiliations:** † Institute of Food Sciences and Technology, 33561 National Taiwan University, Taipei 10617, Taiwan; ‡ Research and Development Department, Syngen Biotech Co., Ltd., Tainan 73055, Taiwan; § Department of Medical Research, China Medical University Hospital, China Medical University, Taichung 40402, Taiwan; ∥ Department of Health and Nutrition Biotechnology, Asia University, Taichung 413305, Taiwan

**Keywords:** sarcopenic obesity, fermented broccoli, gut−muscle axis, Lactiplantibacillus plantarum, metabolomics

## Abstract

Muscle loss accompanied by excessive adiposity is a critical component of metabolic dysfunction. This study investigated the effects of *Lactiplantibacillus plantarum*-fermented broccoli (FHB) in high-fat/high-fructose diet (HFFD)-fed C57BL/6J mice. LC–MS profiling showed that fermentation generated metabolites, including 2-hydroxycaproic acid, 3-phenyllactic acid, and a tetrahydro-β-carboline derivative. FHB supplementation attenuated visceral adiposity, hepatic steatosis, and insulin resistance while preserving grip strength. In the gastrocnemius muscle, FHB was associated with higher phosphorylation of the IRS1/PI3K/Akt and mTOR/p70S6*K*/4E-BP1 pathways and with lower expression of the ubiquitin ligases Atrogin-1 and MuRF-1. 16S rRNA sequencing showed altered gut microbiota, with enrichment of the butyrate-producing species *Anaerostipes hominis* and *Bifidobacterium pseudolongum* in the FHB group. These findings indicate that FHB supplementation was associated with attenuated metabolic dysfunction and preserved muscle function, accompanied by fermentation-derived metabolites and gut microbiota remodeling.

## Graphical abstract

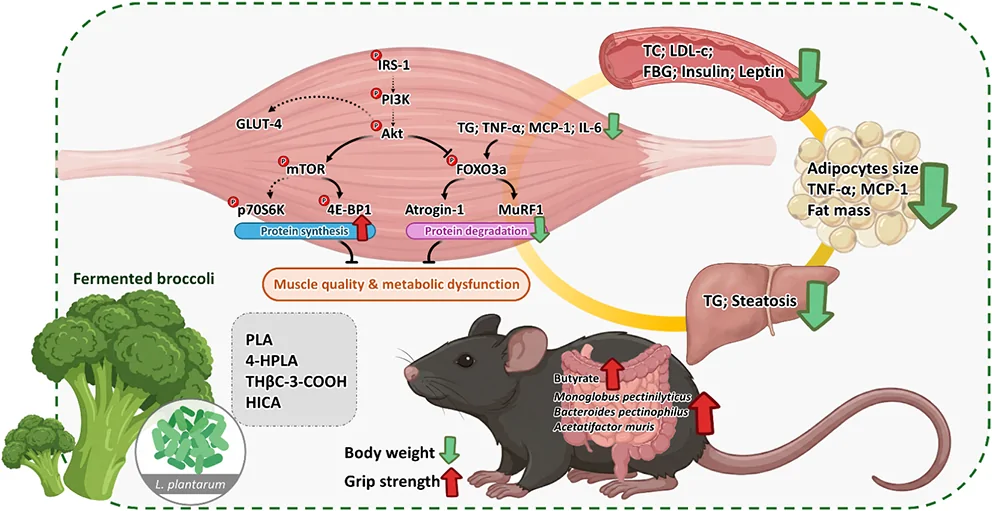

## 1 Introduction

Sarcopenic obesity (SO) represents a complex metabolic syndrome characterized by the simultaneous loss of skeletal muscle and the accumulation of excessive adipose tissue.^1,2^ Unlike sarcopenia or obesity presenting in isolation, the combined phenotype of SO accelerates metabolic dysfunction and significantly increases the risk of cardiovascular disease.^1^ While the etiology of sarcopenic obesity is multifactorial, increasing evidence implicates the western diet high in fat and fructose as a primary risk factor. Chronic consumption of this diet not only promotes visceral adiposity but also induces systemic low-grade inflammation and insulin resistance.^3^ These factors collectively impair muscle protein synthesis, resulting in a pathological phenomenon known as anabolic resistance.^3^ Emerging research has highlighted the critical role of the gut-muscle axis in the pathogenesis of metabolic disorders, including sarcopenic obesity.^4,5^ Dysbiosis of the gut microbiota induced by poor dietary habits compromises the intestinal barrier and elevates circulating levels of pro-inflammatory cytokines such as TNF-α and IL-6.^6,7^ These molecules trigger proteolysis in skeletal muscle via the ubiquitin proteasome pathway.^8,9^ Consequently, therapeutic strategies that could simultaneously restore gut homeostasis and stimulate muscle anabolism are urgently required. Although exercise and protein supplementation are standard interventions, their therapeutic efficacy is frequently compromised by poor patient adherence and the phenomenon of anabolic resistance. Thus, there is a compelling interest in finding functional foods that can modulate the gut microbiota and impart systemic metabolic benefits. Previous studies have suggested that the consumption of cruciferous vegetables is associated with a reduced risk of metabolic syndrome and age-related functional decline.^10,11^ These protective effects are primarily attributed to specific fermentation-derived components, such as isothiocyanates (e.g., sulforaphane) and flavonoids,^12–14^ which have been shown to modulate lipid metabolism and improve insulin sensitivity. Additionally, these phytochemicals may exert myoprotective effects by activating antioxidant defense systems, such as the Nrf2 pathway, and inhibiting NF-κB-mediated inflammatory responses, thereby mitigating the oxidative stress and protein degradation characteristic of sarcopenia.^15^ Broccoli is a rich source of bioactive phytochemicals, particularly glucosinolates and phenolic compounds, that possess antioxidant and anti-inflammatory properties.^16^ However, the therapeutic potential of broccoli is often constrained by the low bioavailability of its native secondary compounds.^17,18^ Fermentation by lactic acid bacteria offers a way to overcome this limitation by enhancing the accessibility and bioactivity of these phytochemicals.^19^ Among the lactic acid bacteria, *Lactiplantibacillus plantarum* is particularly notable for its use in functional food development, owing to its generally recognized as safe (GRAS) status and enzymatic versatility.^20^ Previous studies have demonstrated that lactic acid bacteria, including specific strains of *Lactiplantibacillus plantarum*, function as metabolic converters capable of efficiently biotransforming glucosinolates into bioactive nitriles and other metabolites distinct from those produced by gut bacteria.^19,21^ Furthermore, *Lactiplantibacillus plantarum* can metabolize amino acids into branched-chain hydroxy acids and bioactive peptides. These fermentation-derived compounds may hold specific relevance for muscle preservation by stimulating key anabolic signaling pathways, for example, the effects observed with leucine metabolites such as 2-hydroxycaproic acid.^22,23^ Additionally, organic acids produced during *Lactiplantibacillus plantarum* fermentation, such as 3-phenyllactic acid, have potent antimicrobial properties that may further stabilize the gut microbiota.^24,25^

Despite the known benefits of broccoli and probiotics individually, the synergistic effects of broccoli fermented by *Lactiplantibacillus plantarum* on the complex pathology of sarcopenic obesity remain unexplored. Specifically, it is unclear whether the metabolites generated during fermentation can directly modulate the signaling pathways that govern the muscle mass and lipid metabolism. Therefore, the present study aimed to elucidate the protective effects of *Lactiplantibacillus plantarum*-fermented broccoli in a C57BL/6J mouse model of sarcopenic obesity induced by a high-fat and high-fructose diet. We employed an integrative experimental strategy combining LC-MS metabolomic profiling with comprehensive physiological, histological, and gut microbial analyses to identify key bioactive transformation products and characterize their mechanisms of action along the gut-muscle axis.

## 2 Materials and Methods

### 2.1 Reagents and Antibodies

Antibodies against PI3K, p-PI3K (Tyr458), Akt, p-Akt (Ser473), IRS-1, p-IRS-1 (Tyr612), mTOR, p-mTOR (Ser2448), and p-FoxO3a (Ser318/321) were obtained from Cell Signaling Technology (Beverly, MA, USA). Antibodies against FoxO3a and GLUT4 were purchased from Proteintech (Rosemont, IL, USA), and GAPDH was obtained from Abcam (Cambridge, UK). Antibodies against p70S6K, p-p70S6K (Thr389), 4E-BP1, p-4E-BP1 (Thr37), MuRF1, and Atrogin-1 were obtained from ABclonal (Woburn, MA, USA). The Bio-Rad protein assay dye reagent used for protein quantification was purchased from Bio-Rad Laboratories (Munich, Germany). Xylenes, hematoxylin, and eosin for H&E staining were obtained from Leica Biosystems (Nussloch, Germany). Unless otherwise specified, all other reagents were purchased from Sigma Chemical Co. (St. Louis, MO, USA).

### 2.2 Preparation of Unfermented and Fermented Broccoli Powders

Unfermented and fermented broccoli powders were provided by Syngen Biotech Co., Ltd. (Taiwan) and stored in a dry cabinet until use. Fresh broccoli was washed, trimmed, and blanched in hot water at 80 °C for 30 s. After cooling, the broccoli was freeze-dried and ground to obtain the unfermented broccoli powder. For fermentation, a culture of *Lactiplantibacillus plantarum* SG066 (10^8^ to 10^9^ CFU/mL) was inoculated into the fermentation medium, followed by the addition of broccoli powder. SG066 was selected from an in-house strain collection based on preliminary in vitro screening, in which it showed favorable glucose-regulatory and lipid-lowering activities in HepG2 cell assays (Supplementary Figure S1). The mixture was incubated at 37 °C for 48 h, then freeze-dried, ground into powder, and sterilized to yield the fermented broccoli powder. The final product was plated to determine viable bacterial counts; these were approximately 10^3^ CFU/g. The unfermented and fermented powders were then analyzed for their proximate composition (Supplementary Table S1).

### 2.3 LC–MS Analysis of Metabolites

Metabolite profiling was performed using LC–MS to assess the metabolic changes associated with broccoli and *Lactiplantibacillus plantarum* fermentation. The samples were categorized into medium alone, medium with *Lactiplantibacillus plantarum*, medium with broccoli, and medium with broccoli fermented by *Lactiplantibacillus plantarum*. To determine the specific effects of broccoli fermentation, background signals derived from *Lactiplantibacillus plantarum* alone were subtracted to yield the net broccoli fermentation profile. Chromatographic separation was carried out on an UltiMate 3000 HPLC system (Thermo Fisher Scientific) using a gradient of 0.1% trifluoroacetic acid (TFA) in water (solvent A) and 0.1% TFA in acetonitrile (solvent B), with the following program: 0–10 min, 100% A; 10–40 min, linear gradient to 100% B; 40–50 min, 100% B; and 50.1–60 min, return to 100% A. Mass spectrometry was performed on an Orbitrap Fusion Lumos Tribrid mass spectrometer (Thermo Fisher Scientific) operated in both positive and negative electrospray ionization modes

### 2.4 Animal Experimental Design

Male C57BL/6J mice at 4 weeks of age were obtained from the National Laboratory Animal Center (Taipei, Taiwan). All experimental procedures were approved by the Institutional Animal Care and Use Committee of National Taiwan University (NTU-110-EL-00047). The mice were housed under controlled conditions (25 ± 1 °C, 50% relative humidity, 12 h light/dark cycle) with free access to food and water. After a one-week acclimatization period, the mice were randomly assigned to five groups (*n* = 8 per group): a normal diet (ND), a high-fat high-fructose diet (HFFD), 5% (w/w) unfermented high-dose broccoli (UHB), 1% (w/w) fermented low-dose broccoli (FLB), and 5% (w/w) fermented high-dose broccoli (FHB). Each group was fed for 16 weeks. The ND group received a standard rodent chow diet (LabDiet 5001). The HFFD was based on the D12451 diet (Research Diets, NJ, USA) and supplemented with 20% (v/v) fructose in the drinking water. For the broccoli-supplemented groups, unfermented or fermented broccoli powder was incorporated into the D12451 diet at the expense of an equivalent weight of the base diet (50 g/1000 g for the 5% UHB and FHB groups, and 10 g/1000 g for the 1% FLB group). The composition and caloric distribution of the HFFD-based diets are provided in Supplementary Table S2. At the end of the study, the mice were euthanized with CO_2_ and dissected. Immediately after euthanasia, blood was collected by cardiac puncture and centrifuged to obtain serum. The intestinal tract was then aseptically excised, and its external surface was rinsed with sterile phosphate-buffered saline. Cecal contents were aseptically collected into sterile tubes and stored at −80 °C until short-chain fatty acid analysis. Fecal contents from the colon and rectum were separately collected into sterile tubes and stored at −80 °C until gut microbiota analysis. The liver, visceral adipose tissues, and skeletal muscles were subsequently excised, photographed, weighed, and stored at −80 °C until further analysis. Blood was collected via cardiac puncture, centrifuged to obtain serum, and the organs, including the liver, visceral adipose tissue, and skeletal muscle, were photographed, weighed, and stored at −80 °C until analysis.

### 2.5 Body Composition

Body composition was assessed at week 16 using an ImpediVET bioimpedance spectroscopy analyzer (ImpediMed Ltd., Brisbane, Australia). Each mouse was positioned prone on a padded board, and the body length was measured from the midpoint between the ears to a point 1 cm above the groin. Electrodes were placed at the marked points on the nose and tail. The mouse body weight, length, and age were entered into the instrument to calculate total body water, lean mass, and fat mass.

### 2.6 Grip Strength

Grip strength was measured by using a digital grip strength meter (DigiTech, Japan). The mice were allowed to grasp a metal triangular bar with their forelimbs, and their tails were gently pulled horizontally to keep the body parallel to the table surface until they released the bar. The meter automatically recorded the maximum force at the moment of release. All measurements were performed by an investigator who was blinded to the group assignments. Each mouse was tested three times per day, and the mean values were used for analysis.

### 2.7 Biochemical Evaluation of Serum and Triglyceride Accumulation

Blood samples were centrifuged at 4 °C at 1000×*g* for 10 min, and the serum was stored at −80 °C until analysis. Serum biochemical parameters, including total cholesterol (T-CHO), triglycerides (TG), high-density lipoprotein cholesterol (HDL-c), and low-density lipoprotein cholesterol (LDL-c), were measured by the National Laboratory Animal Center (NLAC, Taipei, Taiwan). Hepatic and muscle tissue triglyceride contents were determined using a commercially available enzymatic colorimetric assay with the absorbance measured at 540 nm, and the values were expressed as milligrams of triglyceride per gram of tissue.

### 2.8 Fasting Blood Glucose (FBG)

The mice were deprived of food for 12 h prior to the testing. After fasting, approximately 0.1 cm of the tail tip was clipped to obtain a blood sample. A drop of blood was applied to a glucose test strip inserted into a glucometer (D&W Blood Glucose Monitoring System, Taiwan), and the fasting blood glucose level was recorded when it was displayed.

### 2.9 Serum Insulin Levels and HOMA-IR and Oral Glucose Tolerance Test (OGTT)

Serum insulin concentrations were determined using a Mouse Insulin ELISA kit (10-1247-01, Mercodia) following the manufacturer’s instructions. At week 16, the mice were fasted for 8 h then given an oral glucose tolerance test. A baseline blood glucose measurement (0 min) was obtained via tail nick, after which glucose was administered orally at 2 g/kg body weight. Blood glucose levels were subsequently measured at 30, 60, 90, and 120 min postadministration. The homeostasis model assessment of insulin resistance (HOMA-IR) was calculated using fasting blood glucose and fasting insulin concentrations according to the following equation:

HOMA‐IR=[fasting glucose(mg/dL)×fasting insulin(mU/mL)]÷405

### 2.10 Serum Leptin and Cytokines

Serum leptin and cytokine concentrations were measured using enzyme-linked immunosorbent assay (ELISA) kits according to the manufacturers’ instructions. Specifically, leptin was quantified using a Mouse Leptin ELISA kit (EZML-82K, Millipore); TNF-α and IL-6 were measured using Mouse Uncoated ELISA kits (Invitrogen), and MCP-1 was measured using the Mouse MCP-1 SimpleStep ELISA kit (ab208979, Abcam). Briefly, target proteins were captured by specific antibodies precoated on 96-well plates, followed by incubation with biotinylated secondary antibodies and horseradish peroxidase (HRP)-conjugated streptavidin. Color development was performed using a tetramethylbenzidine (TMB) substrate, and the absorbance was measured at 450 nm after the reaction was terminated.

### 2.11 Histopathological Examination

Liver, fat, and quadriceps muscle tissues were collected, fixed in 10% formalin, and prepared for histological analysis. Paraffin-embedded tissues were sectioned at 5 μm (liver and fat) or 4 μm (muscle) and stained with hematoxylin and eosin (H&E) to evaluate the cellular morphology. H&E-stained sections were examined for hepatic steatosis and cytoplasmic vacuolation in liver, adipocyte size in perigonadal fat, and myofiber morphology and interstitial spacing in muscle. For lipid accumulation assessment, liver and muscle tissues (0.5 cm × 0.5 cm) were cryoprotected in 30% sucrose, embedded in optimal cutting temperature (OCT) compound, and sectioned at 8 μm for Oil Red O staining. All stained sections were examined under an Olympus IX73 microscope (Olympus, Tokyo, Japan). Oil Red O staining was performed by the Laboratory Animal Center, National Taiwan University College of Medicine (Taipei, Taiwan). Quantification was performed using ImageJ software on three randomly selected fields of view per section. A minimum of 100 adipocytes per animal were measured to calculate the mean cross-sectional area, and adipocyte areas were grouped into size categories (<2500, 2500–5000, 5000–10,000, and >10,000 μm^2^) to determine the size distribution.

### 2.12 Western Blot Analysis

The gastrocnemius muscle was homogenized in ice-cold lysis buffer and incubated on ice for 1 h. The lysates were vortexed intermittently, centrifuged at 12,000×*g* for 30 min at 4 °C, and the supernatants were collected. Protein concentrations were determined using the Bio-Rad protein assay (Bio-Rad, Hercules, CA, USA). Equal amounts of protein (30 μg) were separated on 10% SDS-polyacrylamide gels and transferred onto polyvinylidene difluoride (PVDF) membranes. The membranes were blocked and incubated overnight at 4 °C with primary antibodies diluted according to the manufacturers’ instructions; unless otherwise specified, primary antibodies were used at a dilution of 1:1000. Membranes were then washed and incubated with horseradish peroxidase-conjugated secondary antibodies at a dilution of 1:5000 for 1 h at room temperature. Blots were visualized using a chemiluminescent substrate (Merck Millipore) and imaged with an e-BLOT Touch Imager (e-BLOT Life Science, Shanghai, China). Band intensities were quantified using ImageJ. GAPDH served as the internal loading control.

### 2.13 Next-Generation Sequencing

The composition of the gut microbiota was analyzed as previously described^26,27^ Genomic DNA was extracted and purified using a modified InnuPREP Stool DNA kit, and the samples were submitted to Biotools Co., Ltd. for 16S rRNA amplicon sequencing. The V3–V4 regions of the 16S rRNA gene were amplified by PCR, and sequencing was performed on the Illumina HiSeq 2500 platform (250 bp paired-end reads). The resulting sequences were clustered into OTUs at a 97% identity threshold, representing distinct bacterial taxa at the genus or species level. Microbial community analyses, including α- and β-diversity, were carried out using QIIME (version 1.9.1).

### 2.14 SCFA Analysis

Short-chain fatty acids (SCFAs) were quantified as previously described. Briefly, 0.1 g of feces was mixed with 1 mL of 0.5% phosphoric acid and homogenized twice for 15 s, followed by the addition of 0.5 mL of ethyl acetate and a second round of homogenization (15 s × 2). The mixture was centrifuged at 18,000×*g* for 10 min at 4 °C, and 200 μL of the supernatant was combined with 800 μL of 625 μM 4-methylvaleric acid (an internal standard) to yield a final concentration of 500 μM. The samples were filtered through a 0.22 μm nylon membrane before analysis on an Agilent 7890 Gas Chromatograph System coupled to a 5975 Inert Mass Selective Detector (Agilent Technologies).

### 2.15 Statistical Analysis

Data were analyzed using SPSS software (version 22.0; IBM Corp., Armonk, NY, USA). Group differences were assessed by one-way ANOVA followed by Duncan’s multiple range test. Results are presented as mean ± SEM, and statistical significance was set at *p* < 0.05.

## 3 Results and Discussion

### 3.1 Metabolomic Profiling and Identification of Fermentation-Derived Metabolites

A differential LC–MS analysis was performed to isolate substrate-specific bioconversion products from background signals to identify the metabolic transformations induced by the *Lactiplantibacillus plantarum*-mediated fermentation of broccoli. This targeted profiling revealed a distinct enrichment of eight differentially abundant metabolites (Table 1). The metabolic profile was predominantly characterized by the bioconversion of amino acids into diverse fermentation-derived metabolites capable of counteracting the systemic metabolic stress of sarcopenic obesity. Specifically, fermentation led to the accumulation of several organic acids, including 3-phenyllactic acid (PLA), 2-hydroxycaproic acid (HICA), 4-hydroxyphenyllactic acid (4-HPLA), succinic acid, and threonic acid, that were absent or minor in unfermented broccoli. Concurrently, the analysis identified the β-carboline derivative 2,3,4,9-tetrahydro-1*H*-β-carboline-3-carboxylic acid (THβC-3-COOH), formed via the Pictet–Spengler condensation of tryptophan.^28,29^ Mechanistically, these fermentation-derived aromatic and indole compounds play a critical role in mitigating the pathophysiology of sarcopenic obesity.^30^ Chronic low-grade inflammation and oxidative stress are primary drivers of this condition, which are known to impair insulin signaling and trigger ubiquitin-proteasome-mediated muscle proteolysis.^31^ Both PLA and THβC derivatives have been reported to function as antioxidants and radical scavengers.^24,32,33^ By reducing this oxidative burden, these metabolites may alleviate the systemic inflammation and lipotoxicity that otherwise drive insulin resistance and skeletal muscle wasting, thereby creating a physiological environment conducive to muscle preservation.^30^ The fermentation process yielded a significant generation of 2-hydroxycaproic acid (net abundance 3.5 × 10^8^), a leucine-derived metabolite. Unlike leucine itself, HICA has been demonstrated to exhibit superior anticatabolic properties. Previous studies have indicated that HICA supplementation can stimulate the mTOR signaling pathway to enhance protein synthesis and inhibit metalloproteinase activity, thereby accelerating muscle recovery in models of atrophy.^22,23^ This provides a molecular explanation for the preservation of grip strength and muscle mass observed in the FHB group. Furthermore, the metabolic matrix was enriched with bioactive nitrogenous species, including the cyclic dipeptide cyclo­(phenylalanyl-prolyl) and the tripeptide Phe-Asp-Arg. Cyclic dipeptides are increasingly being acknowledged as stable signaling molecules capable of crossing the intestinal barrier,^25^ suggesting that they may facilitate systemic communication along the gut–muscle axis. The results indicate that *Lactiplantibacillus plantarum* fermentation transforms broccoli into a functional metabolic modulator that synergistically targets the inflammatory, oxidative, and catabolic drivers of sarcopenic obesity.

**Table 1:** Differentially Abundant Metabolites before and after Fermentation of Broccoli by *Lactiplantibacillus plantarum* ^a^,^b^

No.	Name	Formula	Calcd MW	*m*/*z*	Area (max.)	Area 1: Med	Area 2: Med+*Lac*	Area 3: Med+Br	Area 4: Med + Br + *Lac*	Area: (4–3)-(2–1)
1	l-(−)-3-Phenyllactic acid	C_9_H_10_O_3_	166.063	165.056	1,677,086,445	1.4 × 10^7^	1.1 × 10^9^	1.3 × 10^7^	2.0 × 10^9^	9.0 × 10^8^
2	Cyclo(phenylalanyl-prolyl)	C_14_H_16_N_2_O_2_	262.131	263.139	402,826,631	2.9 × 10^7^	2.6 × 10^7^	2.3 × 10^7^	4.0 × 10^8^	3.8 × 10^8^
3	2-Hydroxycaproic acid	C_6_H_12_O_3_	132.078	131.071	1,039,765,692	4.0 × 10^7^	6.9 × 10^8^	4.3 × 10^7^	1.0 × 10^9^	3.5 × 10^8^
4	dl-4-Hydroxyphenyllactic acid	C_9_H_10_O_4_	182.058	181.050	345,561,479	1.3 × 10^6^	2.2 × 10^8^	1.1 × 10^7^	3.5 × 10^8^	1.2 × 10^8^
5	2,3,4,9-Tetrahydro-1*H*-β-carboline-3-carboxylic acid	C_12_H_12_N_2_O_2_	216.0896	217.0969	114,458,664	N.D.	N.D.	6.8 × 10^6^	1.1 × 10^8^	1.1 × 10^8^
6	Phe-Asp-Arg	C_19_H_28_N_6_O_6_	454.218	455.225	116,124,421	3.0 × 10^7^	4.8 × 10^7^	3.6 × 10^7^	1.2 × 10^8^	6.3 × 10^7^
7	l-Threonic acid	C_4_H_8_O_5_	136.038	135.031	74,849,193	5.8 × 10^6^	2.9 × 10^7^	5.3 × 10^6^	7.5 × 10^7^	4.6 × 10^7^
8	Succinic acid	C_4_H_6_O_4_	118.026	117.019	86,516,951	2.0 × 10^7^	6.8 × 10^7^	3.2 × 10^7^	8.7 × 10^7^	6.0 × 10^6^

a Metabolite intensities were obtained via Orbitrap Fusion Lumos mass spectrometry in both positive and negative ion modes . The metabolites were identified and annotated using Compound Discoverer.

b Metabolomic profiles were compared across four experimental conditions to evaluate the impact of broccoli fermentation with Lactiplantibacillus plantarum. Area (1) represents the plain culture medium; Area (2) corresponds to the medium inoculated with L. plantarum; Area (3) includes the medium with unfermented broccoli, and Area (4) reflects the fermented broccoli condition after incubation with *Lactiplantibacillus plantarum*. The contrast [(Area4 – Area3) – (Area2 – Area1)] was calculated to identify the metabolite changes specifically attributable to the fermentation of broccoli. This differential metric eliminates background effects from the medium and bacterial inoculation alone, highlighting compounds that are uniquely elevated or suppressed due to the interaction between broccoli and *Lactiplantibacillus plantarum* during fermentation. N.D., not detected.

### 3.2 Effects of Fermented Broccoli on Body Weight and Metabolic Parameters in HFFD-Fed Mice

To assess the physiological impact of broccoli fermentation under a high-fat, high-fructose diet (HFFD), C57BL/6J mice were administered unfermented or fermented broccoli extracts over a 16-week period (Figure 1A). At the study’s end point, mice in the HFFD group exhibited visually apparent abdominal distension and a rounded morphology, compared to the leaner appearance of normal diet (ND) mice (Figure 1B). All three broccoli interventions (UHB, FLB, and FHB) significantly mitigated weight gain relative to the HFFD group, with no significant differences among them (Figure 1C). Total body weight gain in the HFFD group reached 22.8 ± 2.1 g, significantly higher than that of all other groups (Figure 1D). Food intake did not differ among the four HFFD-based groups (HFFD, UHB, FLB, and FHB); although these groups consumed less food by weight than the ND group, their daily caloric intake was comparable to or slightly higher than that of the ND group owing to the higher energy density of the HFFD, and the broccoli-supplemented groups showed a lower energy efficiency ratio than the HFFD group (Supplementary Table S3). Waist circumference measurements showed that the HFFD group had the broadest average waistline (6.42 ± 0.2 cm). Both FLB (5.88 ± 0.4 cm) and FHB (5.84 ± 0.3 cm) groups exhibited significantly smaller waistlines compared to the HFFD-fed mice, indicating a partial protective effect against central adiposity (Figure 1E) (Supplementary Table S4). Body composition analysis via InBody further highlighted substantial alterations in the distribution of fat and lean mass. The HFFD group displayed a significantly elevated fat mass-to-body weight ratio (54.6 ± 4.7%) compared to the ND group (34.4 ± 9.8%), accompanied by a reduction in lean mass (HFFD: 45.4 ± 4.7% vs ND: 65.6 ± 9.8%). Broccoli supplementation ameliorated these imbalances in a fermentation- and dose-dependent manner. The FHB group exhibited the most favorable composition, with the fat mass reduced to 41.7 ± 7.2% and lean mass increased to 58.3 ± 7.2%, approaching levels seen in the ND group (Figure 1F–G). In terms of serum biochemical markers, HFFD-fed mice exhibited significantly elevated levels of triglycerides (TG), total cholesterol (T-CHO), low-density lipoprotein cholesterol (LDL-C), and disturbed lipid ratios, including T-CHO/HDL-C, indicative of dyslipidemia under metabolic stress. Broccoli supplementation reduced serum total cholesterol and LDL-C levels relative to the HFFD group (Figure 1H–L).

**Figure 1:**
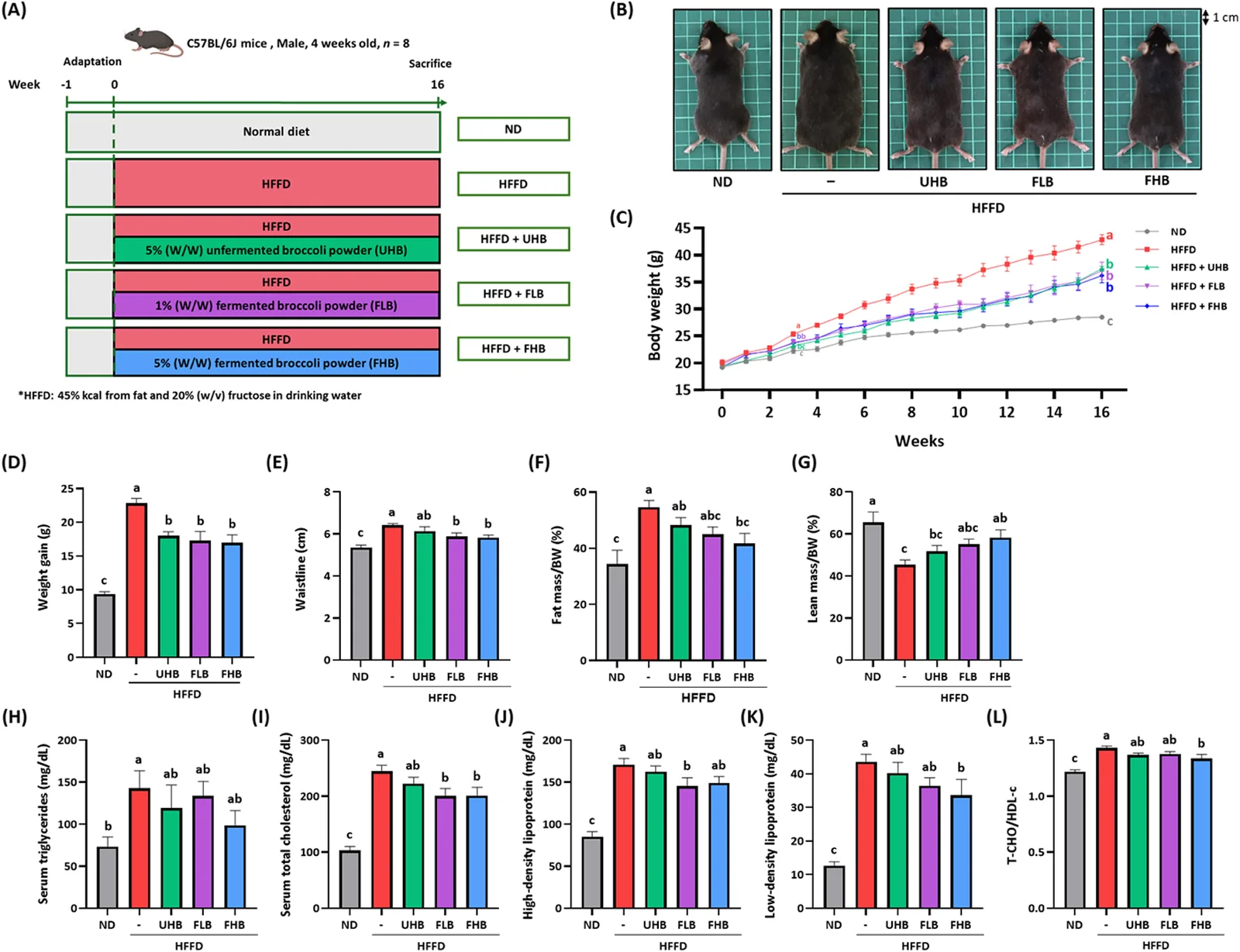
Effects of fermented broccoli on body weight, food intake, and serum lipid profiles in HFFD-fed C57BL/6J mice. (A) Experimental design and feeding regimen. (B) Representative gross morphology of mice at the study end point. (C) The weekly progression in body weight. (D) Total body weight gain. (E) Waistline measurement. (F) Fat mass-to-body weight ratio. (G) Lean mass-to-body weight ratio. (H–L) Serum biochemical parameters, including triglycerides, total cholesterol, low-density lipoprotein (LDL), high-density lipoprotein (HDL), and the total cholesterol to HDL-cholesterol ratio (T-CHO/HDL-c). Data are presented as means ± SEM. Different letters (a–c) indicate significant differences among groups (*p* < 0.05).

### 3.3 Effects of Fermented Broccoli on Hepatic Lipid Accumulation in HFFD-Fed Mice

Liver, kidney, and spleen were collected and evaluated at the study end point to assess the protective effect of fermented broccoli against hepatic steatosis (Figure 2A). Livers from HFFD-fed mice appeared enlarged and pale, indicating lipid infiltration, whereas those from the FHB group exhibited a darker hue, similar to the ND group. Quantitative measurements confirmed that HFFD feeding significantly increased liver weight (1.93 ± 0.24 g) compared to ND (1.38 ± 0.20 g), while supplementation with unfermented (UHB) or fermented broccoli (FLB, FHB) significantly reduced liver weight relative to the HFFD group, with no significant differences among the supplemented groups (Figure 2B). Kidney and spleen weights were also significantly elevated in the HFFD group compared to the controls, whereas supplementation with broccoli, particularly in the FHB group, mitigated these changes (Figure 2C, D), indicating a partial attenuation of systemic organ hypertrophy. The absolute and body-weight-normalized weights of the liver, kidney, spleen, adipose depots, and skeletal muscles are compiled in Supplementary Table S5. Biochemical analysis revealed that hepatic TG levels were significantly elevated in HFFD-fed mice (7.50 ± 1.17 mg/g liver), confirming the induction of steatosis (Figure 2E). Both UHB and FHB supplementation (UHB: 5.41 ± 0.33 mg/g liver; FHB: 5.24 ± 0.50 mg/g liver) reduced hepatic TG accumulation, approaching the levels observed in ND mice. Histological examination further supported these findings: H&E staining showed microvesicular lipid droplet accumulation in the HFFD group, whereas the fermented broccoli treatment attenuated vacuolar degeneration (Figure 2F). Oil Red O staining corroborated this improvement, revealing reduced lipid droplet size and density, particularly in pericentral regions (Figure 2G).

**Figure 2:**
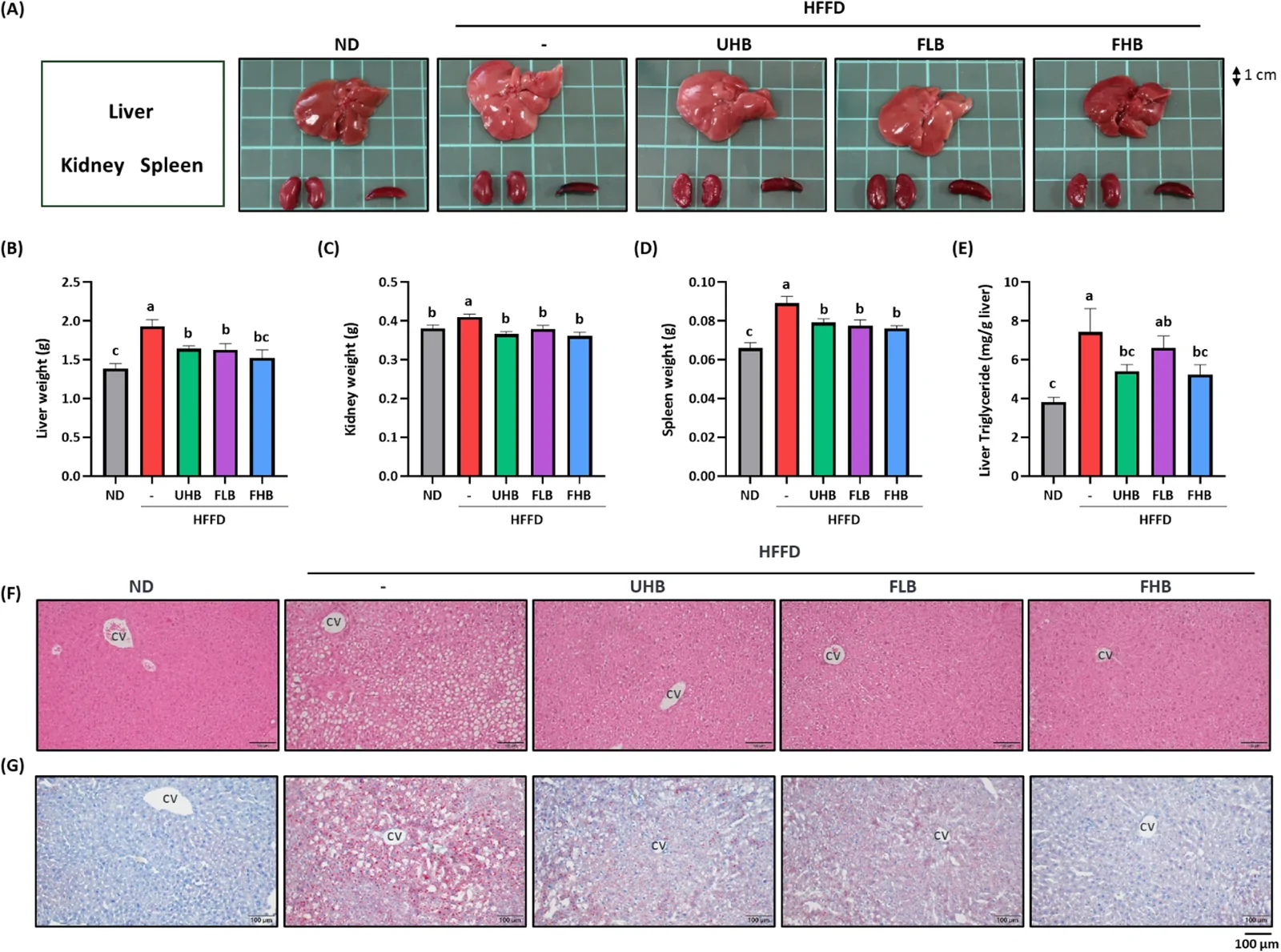
Effects of fermented broccoli on liver weight and hepatic triglyceride accumulation in HFFD-fed C57BL/6J mice. (A) Representative images of the liver, kidney, and spleen from each group at the study end point. Organ weights of (B) liver, (C) kidney, and (D) spleen are shown. (E) Hepatic triglyceride content (*n* = 6–8 per group). (F) Representative hematoxylin and eosin (H&E) staining, and (G) Oil Red O staining of liver tissue sections. CV, central vein. All histological images were captured at 200× magnification. Scale bars = 100 μm (*n* = 3 per group). Data are presented as means ± SEM. Different letters (a–c) indicate significant differences among groups (*p* < 0.05).

### 3.4 Effects of Fermented Broccoli on Visceral Adiposity, Adipocyte Hypertrophy, and Inflammatory Cytokines in HFFD-Fed Mice

Visceral obesity is a hallmark of metabolic syndrome, contributing to systemic inflammation and insulin resistance. Therefore, we assessed the impact of fermented broccoli supplementation on visceral fat accumulation and adipose tissue inflammation in mice fed the HFFD. Mice in the HFFD group developed perigonadal and retroperitoneal fat depots compared to those in the ND group, as visually evident and confirmed by weight measurements (Figure 3A–D). Compared to the HFFD group, UHB, FLB, and FHB interventions significantly reduced the weights of perigonadal, retroperitoneal, and total visceral adipose tissue, defined as the sum of the perigonadal and retroperitoneal fat depots, (*p* < 0.05), suggesting their effectiveness in alleviating diet-induced fat accumulation (Supplementary Table S5).

**Figure 3:**
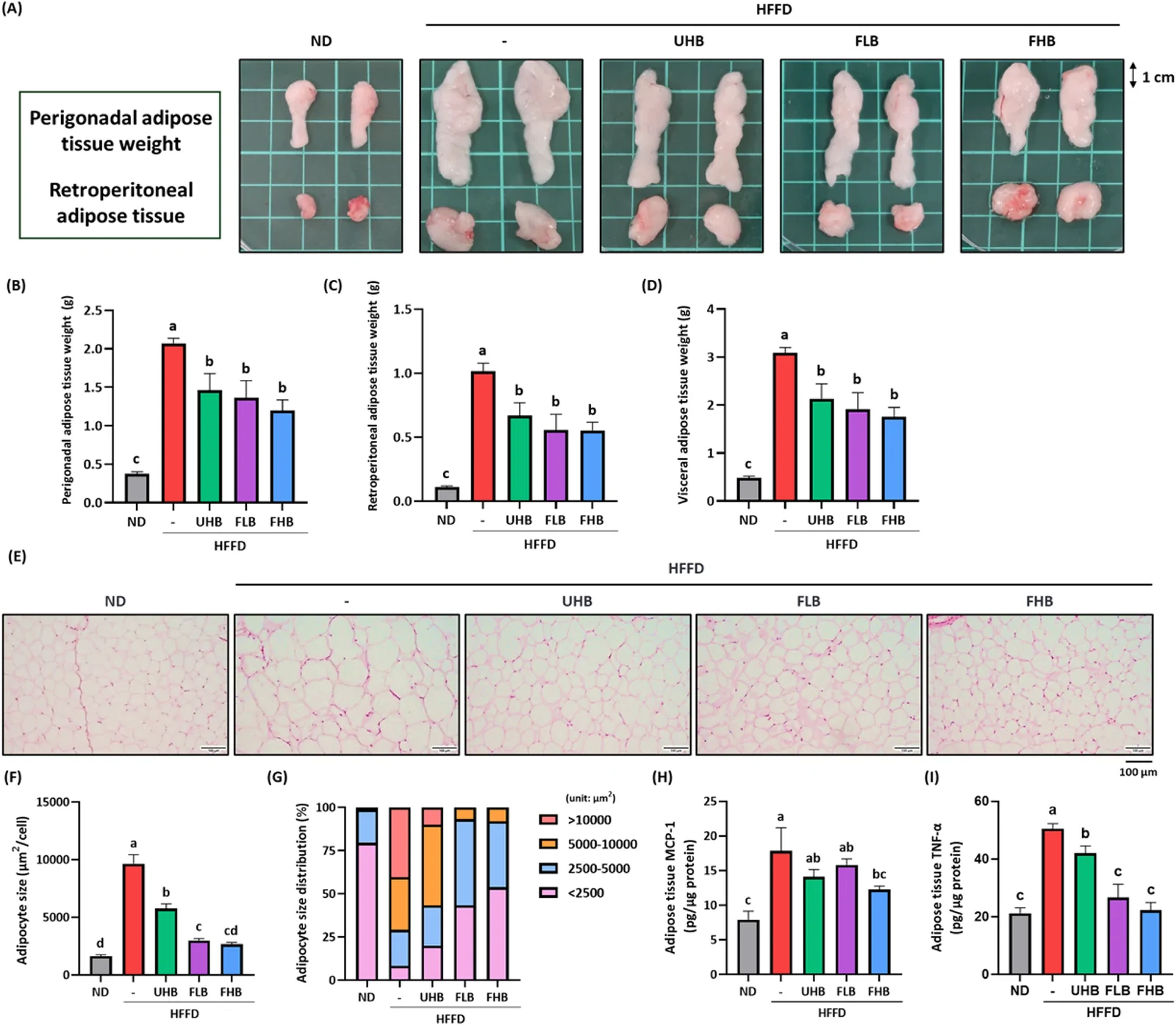
Effects of fermented broccoli on visceral adiposity, adipocyte hypertrophy, and inflammatory cytokine levels in HFFD-fed C57BL/6J mice. (A) Representative images of perigonadal and retroperitoneal adipose tissue from each group. Weights of (B) perigonadal, (C) retroperitoneal, and (D) total visceral adipose tissue (sum of perigonadal and retroperitoneal fat) were recorded (*n* = 8 per group). (E) Representative hematoxylin and eosin (H&E) staining of perigonadal adipose tissue sections. (F) Quantification of average adipocyte size and (G) adipocyte size distribution, based on ImageJ analysis (*n* = 4 per group). (H, I) Levels of MCP-1 and TNF-α in adipose tissue homogenates (*n* = 5 per group). Scale bars = 100 μm. Data are presented as means ± SEM. Different letters (a–d) indicate significant differences among groups (*p* < 0.05).

Histological analysis further revealed marked adipocyte hypertrophy in the HFFD group (Figure 3E). Quantification of adipocyte size showed significant enlargement in HFFD mice (Figure 3F), which was partially reversed by UHB and further attenuated by FHB supplementation. The distribution of adipocyte size (Figure 3G) indicated a shift toward smaller adipocytes in the FHB group, highlighting improved adipose remodeling following fermentation. The levels of MCP-1 and TNF-α were measured to assess the degree of inflammation in adipose tissue (Figure 3H, I). Both cytokines were significantly elevated in the HFFD group compared to the ND controls. FHB supplementation effectively reduced MCP-1 levels. TNF-α levels were markedly suppressed in all broccoli-fed groups (UHB, FLB, and FHB), suggesting a consistent anti-inflammatory effect regardless of fermentation status. These findings indicate that fermented broccoli not only limits visceral adiposity but also mitigates inflammation in adipose tissue, a factor that plays a critical role in preventing obesity-related metabolic dysfunction.

### 3.5 Effects of Fermented Broccoli on Hyperglycemia and Insulin Resistance in HFFD-Fed Mice

Excessive adiposity is tightly associated with impaired glucose homeostasis and insulin resistance, two hallmarks of metabolic syndrome. Fasting blood glucose, insulin levels, HOMA-IR index, and oral glucose tolerance were assessed to determine whether fermented broccoli confers glycemic benefits under obesogenic stress. By week 4, fasting glucose in HFFD-fed mice was already significantly elevated compared to the ND group, and this hyperglycemia persisted throughout the study (week 16: ND: 110.1 ± 12.3 mg/dL; HFFD: 146.3 ± 10.3 mg/dL). Broccoli supplementation significantly lowered fasting glucose compared to HFFD controls, most clearly in the FLB and FHB groups (FHB: 125 ± 15.7 mg/dL) (Figure 4A). Fasting and postprandial insulin levels were markedly increased in the HFFD group, indicative of insulin resistance. The fermented broccoli groups (FLB and FHB) significantly reduced both fasting and postprandial insulin relative to HFFD controls, whereas UHB did not significantly lower fasting insulin. For postprandial insulin, the FHB group showed the lowest level among the supplemented groups (Figure 4B, C). Consistent with this result, the HOMA-IR index—a surrogate for insulin resistance—was significantly reduced in the FHB group compared to both the HFFD and UHB groups, suggesting enhanced insulin sensitivity through fermentation (Figure 4D). Serum leptin levels, a marker of adiposity and leptin resistance, were significantly elevated in the HFFD group (5.41 ± 0.05 ng/mL vs ND: 0.24 ± 0.09 ng/mL). FHB supplementation markedly reduced leptin to 2.27 ± 0.7 ng/mL, while FLB also showed a significant reduction (1.98 ± 0.86 ng/mL), supporting the antiobesogenic and metabolic benefits of fermented broccoli (Figure 4E). Glucose tolerance was evaluated by oral glucose tolerance tests (OGTT) at weeks 7 and 15. HFFD-fed mice exhibited impaired glucose clearance, with elevated blood glucose persisting at 60, 90, and 120 min postglucose load (Figure 4F–I). Although AUC analyses did not reveal a statistically significant difference, FHB supplementation exhibited a trend toward improved glucose tolerance over time compared to the HFFD group. Taken together, these findings suggest that fermented broccoli mitigates diet-induced glucose dysregulation, insulin resistance, and leptin elevation, highlighting its potential in managing obesity-related metabolic disorders.

**Figure 4:**
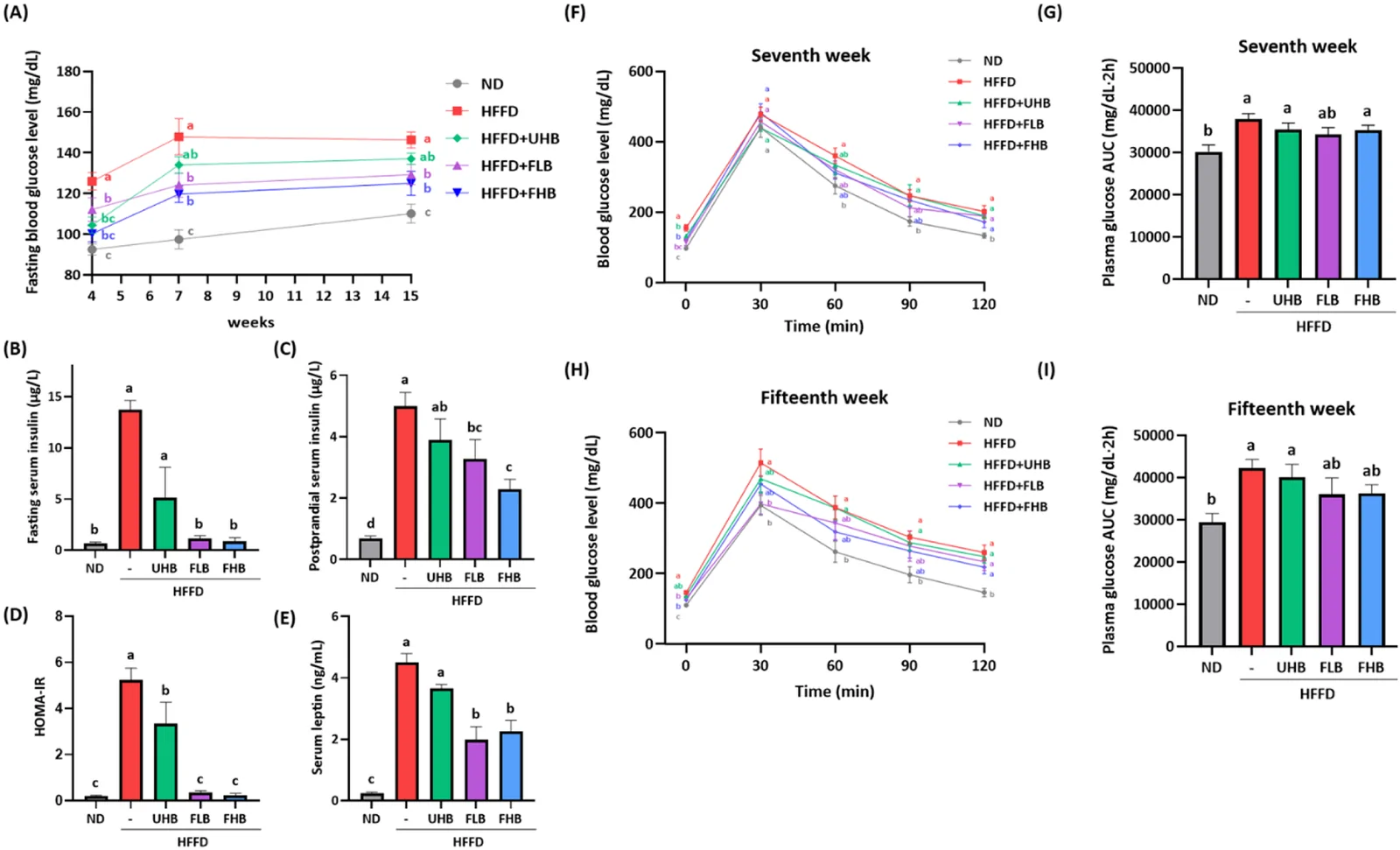
Effects of fermented broccoli on fasting glucose, insulin, and glucose tolerance in HFFD-fed C57BL/6J mice. Blood glucose was monitored at the indicated time points using a glucometer. (A) Fasting blood glucose levels. (B) Fasting serum insulin concentrations. (C) Postprandial serum insulin concentrations (*n* = 7 per group). (D) HOMA-IR (*n* = 3 per group). (E) Serum leptin concentrations (*n* = 4 per group). (F) Oral glucose tolerance test (OGTT) curves at week 7. (G) The corresponding area under the curve (AUC) at week 7. (H) OGTT curves at week 15. (I) The corresponding AUC at week 15 (*n* = 7 per group). Data are expressed as mean ± SEM. Different letters (a–c) indicate significant differences among groups (*p* < 0.05).

### 3.6 Effects of Fermented Broccoli on Muscle Function and Intramuscular Lipid Content in HFFD-Fed Mice

To evaluate the impact of fermented broccoli on skeletal muscle health under metabolic stress, we assessed grip strength, muscle mass, histomorphology, intramuscular lipid content, and inflammatory markers in HFFD-fed mice. At week 9, the HFFD group displayed a significant reduction in grip strength compared to the ND controls, indicating impaired muscle function. Broccoli supplementation significantly restored grip strength relative to the HFFD group, and this improvement was sustained through week 13, with no significant differences among the supplemented groups (Figure 5A). Histological evaluation and tissue imaging revealed comparable gross muscle morphology across groups (Figure 5B). Analysis of normalized muscle weights (expressed as mg of muscle per g of body weight) revealed that HFFD feeding significantly reduced the normalized mass of the quadriceps, gastrocnemius, and soleus relative to the ND group (Figure 5C–E) (Supplementary Table S5). In the gastrocnemius, FLB and FHB restored normalized mass to levels comparable to the ND group and significantly higher than HFFD controls, whereas UHB remained comparable to HFFD. A similar pattern was observed in the soleus, where FHB restored normalized mass to the ND level. Histopathological examination with H&E staining revealed muscle fiber atrophy and disrupted morphology in the HFFD group, accompanied by lipid infiltration visualized with Oil Red O staining (Figure 5F, G). Intramuscular triglyceride (TG) accumulation was also markedly increased in the HFFD group. All broccoli-treated groups (UHB, FLB, and FHB) significantly reduced TG content relative to the HFFD group, with the FLB and FHB groups approaching the levels observed in ND controls (Figure 5H). Pro-inflammatory cytokines in skeletal muscle were markedly elevated in the HFFD group, reflecting a state of diet-induced inflammation. TNF-α levels were significantly higher in HFFD-fed mice than in ND controls, while broccoli supplementation significantly suppressed TNF-α expression relative to the HFFD group, with no significant differences among the supplemented groups (Figure 5I). A similar pattern was observed for IL-6, where the levels were significantly increased in the HFFD group but significantly attenuated by all broccoli interventions (Figure 5J). MCP-1 levels were also elevated in the HFFD group; however, both FLB and FHB exhibited significantly lower MCP-1 expression compared to the HFFD group (Figure 5K). These results indicate that HFFD feeding impaired muscle quality, as reflected by reduced grip strength, intramuscular lipid accumulation, and elevated pro-inflammatory cytokines, and that broccoli supplementation attenuated these changes.

**Figure 5:**
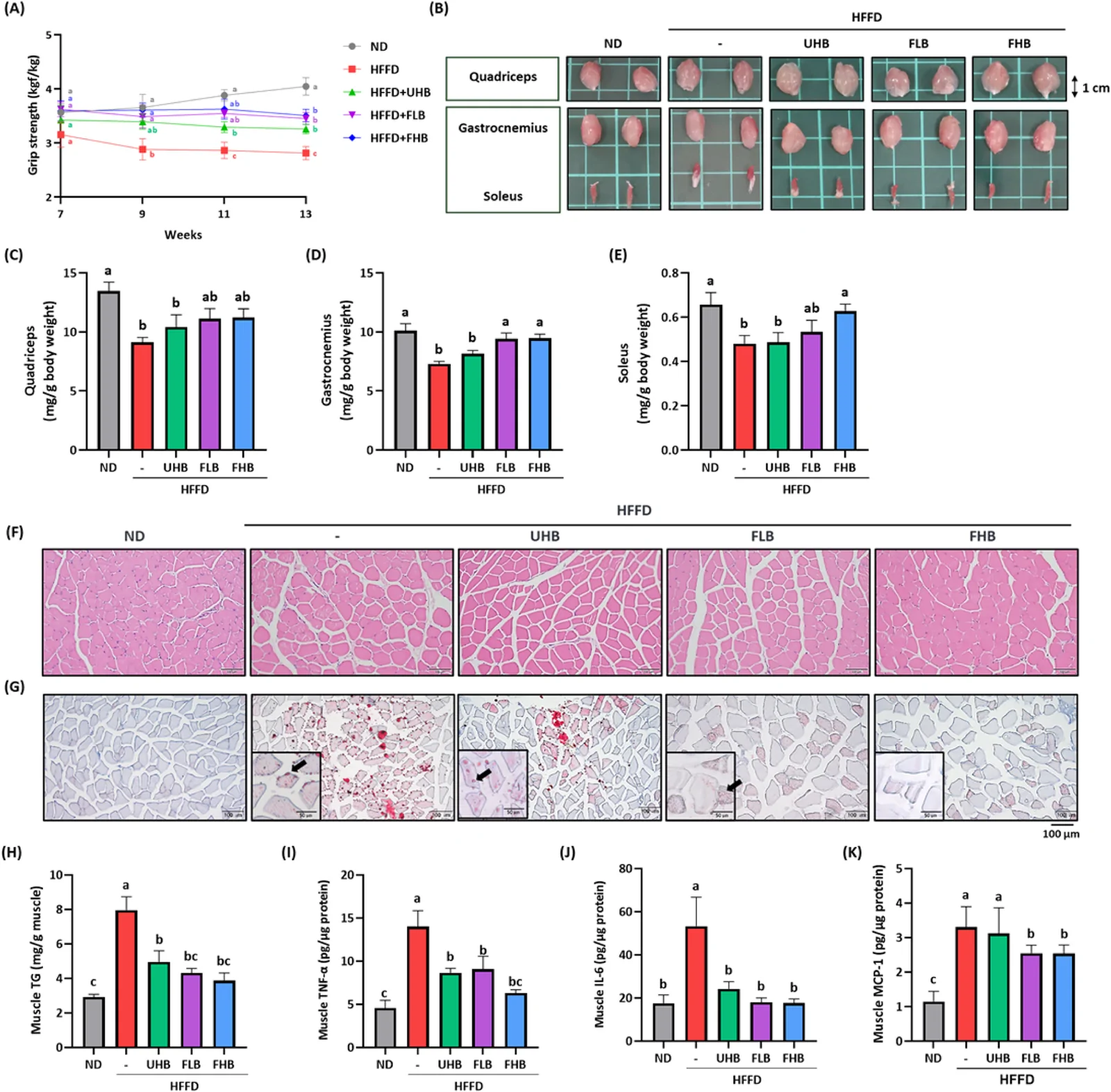
Effects of fermented broccoli on muscle function and intramuscular lipid content in HFFD-fed C57BL/6J mice. (A) Grip strength. (B) Representative images of quadriceps, gastrocnemius, and soleus muscles at the time of sacrifice. (C–E) Normalized muscle weights of quadriceps, gastrocnemius, and soleus relative to body weight (*n* = 8 per group). (F) Hematoxylin and eosin (H&E) staining of muscle cross sections. (G) Oil Red O staining of muscle cross sections showing lipid deposition; the insets highlight representative lipid droplets. Scale bars = 100 μm. (H) Intramuscular triglyceride content (*n* = 5 per group). (I–K) Protein levels of TNF-α, IL-6, and MCP-1 in skeletal muscle. Data are expressed as mean ± SEM. Different letters (a–c) indicate significant differences among groups (*p* < 0.05).

### 3.7 Effects of Fermented Broccoli on Muscle Protein Synthesis and Degradation Signaling in HFFD-Fed Mice

We examined key regulators of protein synthesis and degradation pathways in the gastrocnemius muscle to explore the molecular mechanisms associated with the observed changes in muscle quality. Mice fed an HFFD exhibited marked suppression of the IRS1/PI3K/Akt signaling cascade, as indicated by significantly reduced phosphorylation of IRS1, PI3K, and Akt, along with decreased GLUT4 expression (Figure 6A). FHB supplementation restored this signaling axis by enhancing the phosphorylation of IRS1, PI3K, and Akt, as well as increasing GLUT4 levels, suggesting improved insulin sensitivity and glucose uptake capacity in the gastrocnemius muscle. Additionally, the phosphorylation of key proteins in the mTOR pathway, including mTOR, 4E-BP1, and p70S6K, was significantly reduced in HFFD-fed mice, indicating impaired anabolic signaling (Figure 6B). Both UHB and FHB supplementation significantly restored the phosphorylation levels of all three targets relative to HFFD controls, with FHB showing significantly greater restoration of p-mTOR than UHB, while p-4E-BP1 and p-p70S6K did not differ significantly between the two. These findings indicate that both unfermented and fermented broccoli were associated with the attenuation of HFFD-induced suppression of mTOR signaling in the gastrocnemius muscle. In addition, we evaluated the markers of protein degradation. HFFD significantly reduced FOXO3a phosphorylation while elevating the expression of its downstream E3 ligases, atrogin-1 and MuRF-1, consistent with enhanced proteolysis^34,35^ (Figure 6C). FHB supplementation significantly restored FOXO3a phosphorylation and reduced MuRF-1 expression relative to both HFFD and UHB, whereas atrogin-1 was reduced comparably by UHB and FHB. Overall, these results indicate that broccoli supplementation was associated with improved anabolic signaling capacity and reduced catabolic signaling in the gastrocnemius muscle under obesogenic conditions, with FHB showing significantly greater improvements than UHB in p-Akt, GLUT4, p-mTOR, FOXO3a phosphorylation, and MuRF-1.

**Figure 6:**
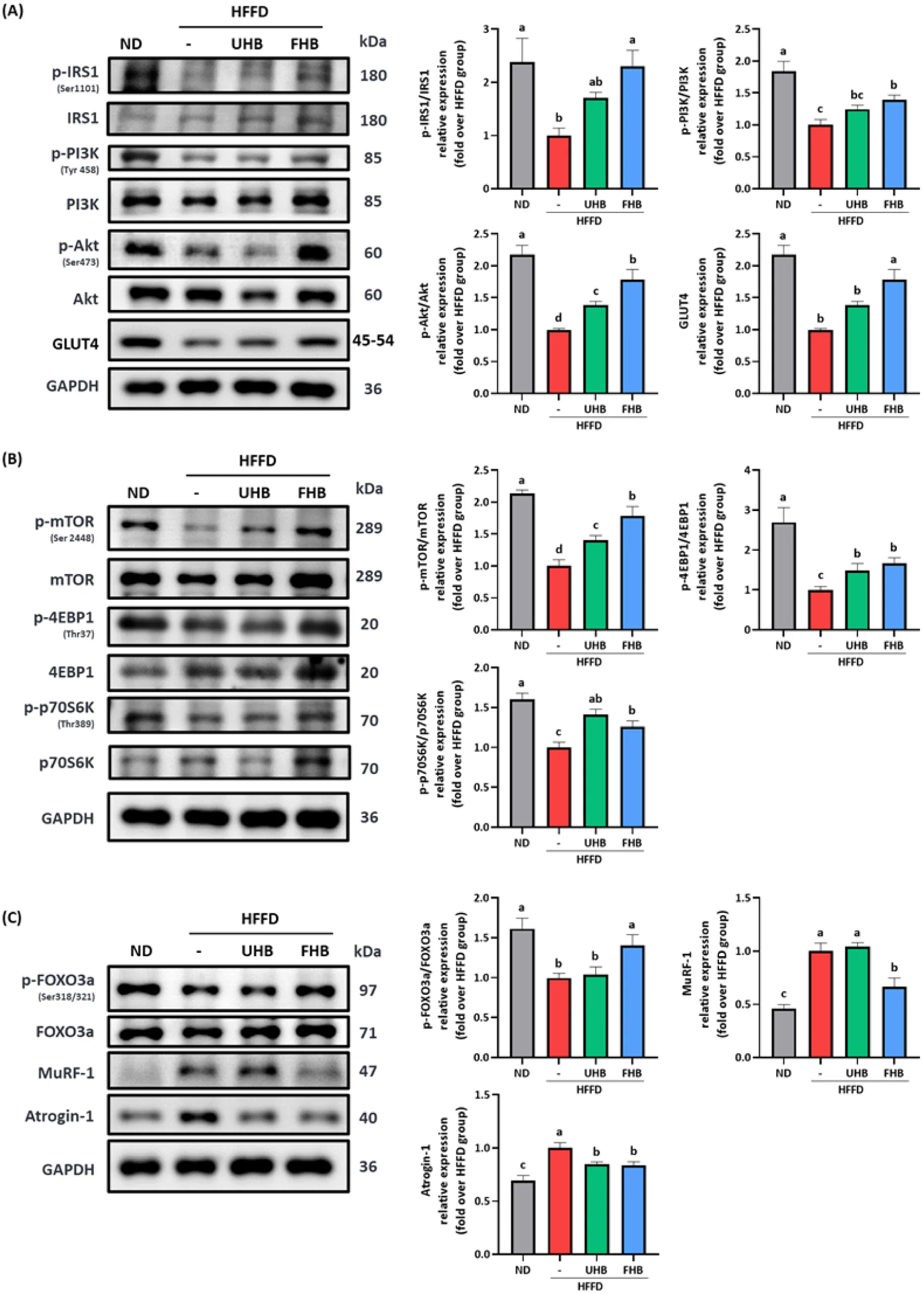
Effects of fermented broccoli on protein synthesis and degradation signaling in gastrocnemius muscle of HFFD-fed C57BL/6J mice. (A) Representative western blots and quantification of p-IRS1/IRS1, p-PI3K/PI3K, p-Akt/Akt, and GLUT4 protein expression. (B) Representative western blots and quantification of p-mTOR/mTOR, p-4E-BP1/4E-BP1, and p-p70S6K/p70S6K. (C) Representative western blots and quantification of p-FOXO3a/FOXO3a, MuRF-1, and atrogin-1. Data are expressed as mean ± SEM (*n* = 4 per group). Different letters (a–d) indicate significant differences among groups (*p* < 0.05).

These fermentation-derived metabolites, rather than broccoli’s native phytochemicals, appear to distinguish the fermented product. Whereas broccoli-derived sulforaphane has been reported to suppress muscle inflammation via antioxidant pathways,^12,15^ fermentation did not significantly alter the proximate composition (protein, fat, and dietary fiber) (Supplementary Table S1) or the total flavonoid, polyphenol, or isothiocyanate content (Supplementary Figure S2), indicating that the compositional difference lies in the newly formed metabolites. This distinction may also explain the differential effects noted between the two preparations. Body weight gain and hepatic steatosis, which are governed largely by fiber and caloric intake, were improved comparably by the unfermented and fermented groups, consistent with their similar fiber and macronutrient contents. In contrast, the end points on which the fermented group showed a significantly greater effect, such as postprandial insulin and muscle p-Akt, GLUT4, p-mTOR, and MuRF-1, correspond to pathways that may be more responsive to the fermentation-derived metabolites (Table 1). We propose this as a hypothesis consistent with the present data.

### 3.8 Effects of Fermented Broccoli on Gut Microbiota Composition and Fecal SCFAs

The gut–muscle axis has been increasingly recognized as an important component of the metabolic and inflammatory disturbances associated with sarcopenia and sarcopenic obesity, in which diet-induced gut dysbiosis may influence skeletal muscle function through microbial metabolites, impaired intestinal barrier integrity, systemic inflammation, and altered insulin sensitivity.^4,5,36^ We therefore analyzed the fecal microbiota by 16S rRNA sequencing. No statistically significant differences were observed in the α-diversity indices among the experimental groups. Nevertheless, the FHB group showed numerically higher observed features, Shannon entropy, and Margalef richness than the HFFD group, whereas Pielou’s evenness remained comparable among groups (Supplementary Figure S3). The present data therefore do not demonstrate a significant restoration of α-diversity but indicate a numerical tendency toward greater microbial richness following FHB supplementation. t-Distributed stochastic neighbor embedding (t-SNE) analysis showed distinct clustering patterns among the experimental groups, with the FHB samples separated from the HFFD samples, indicating differences in overall microbial community structure (Figure 7A). Because t-SNE is primarily a nonlinear dimensionality reduction and visualization method, this separation should not be interpreted as statistical evidence of altered β-diversity without a corresponding multivariate test such as PERMANOVA.^37^ Species-level relative abundance analysis and hierarchical clustering also demonstrated distinct microbial profiles among the groups (Figure 7B, C). The HFFD group showed relatively higher abundances of *Mucispirillum schaedleri* and taxa assigned to Deferribacteres. *M. schaedleri* colonizes the intestinal mucus layer and has been associated with inflammatory conditions in several mouse models, and in severely immunodeficient mice, it has been causally linked to the development of Crohn’s disease-like colitis.^38–40^ However, *M. schaedleri* can exert context-dependent effects, including protection against Salmonella-induced colitis under some conditions.^40^ Its higher abundance in the HFFD group should therefore be interpreted as an HFFD-associated microbial alteration rather than definitive evidence of pathogenic activity.

**Figure 7:**
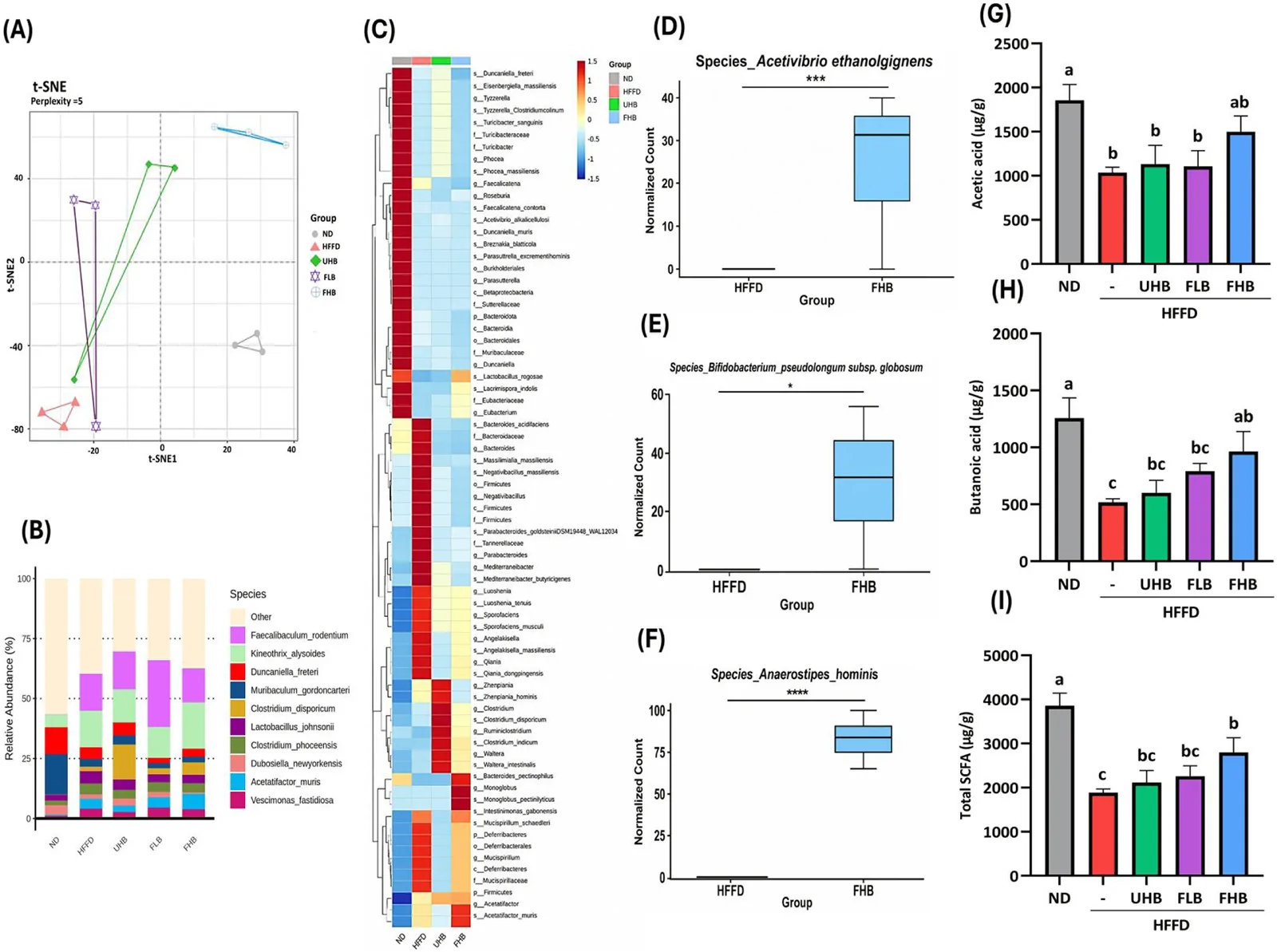
Effects of fermented broccoli on gut microbiota composition and fecal short-chain fatty acid concentrations in HFFD-fed mice. (A) t-SNE visualization of gut microbial community structure among the experimental groups. (B) Relative abundance of predominant bacterial species. (C) Hierarchical clustering heatmap of differentially abundant bacterial taxa. The color scale represents normalized abundance expressed as Z-scores. (D–F) Normalized abundance of *Acetivibrio ethanolgignens*, *Bifidobacterium pseudolongum* subsp. *globosum*, and *Anaerostipes hominis* in the HFFD and FHB groups. (G–I) Fecal concentrations of acetic acid, butanoic acid, and total SCFAs. Data are expressed as mean ± SEM. Different letters indicate significant differences among groups at *p* < 0.05. Asterisks indicate significant differences between the indicated groups (*p* < 0.05, **p < 0.001, and ***p < 0.0001).

In contrast, pairwise differential-abundance analysis showed that *Acetivibrio ethanolgignens*, *Bifidobacterium pseudolongum* subsp. *globosum*, and *Anaerostipes hominis* were significantly more abundant in the FHB group than in the HFFD group (Figure 7D–F). Among these, *A. hominis* has been characterized as a butyrate-producing species,^41^ and its enrichment was biologically consistent with the significantly higher fecal butanoic acid concentration observed in the FHB group. *B. pseudolongum* has been reported to reduce circulating triglycerides, visceral adiposity, and energy intake while remodeling the gut microbiota in high-fat diet-fed mice,^42^ and its higher abundance was consistent with the improved adiposity and metabolic phenotypes following FHB supplementation. *A. ethanolgignens*, which has been reclassified as *Acetanaerobacter ethanolgignens*,^43^ was also significantly enriched; as direct evidence linking this taxon to skeletal muscle metabolism remains limited, its enrichment is regarded as a differential microbial characteristic of the FHB group rather than a confirmed functional mediator. Additional differential taxa analyses showed that *Monoglobus pectinilyticus* and members of *Roseburia* were also enriched in the FHB group and are presented in the Supporting Information. *M. pectinilyticus* is a specialized pectin-degrading bacterium with a highly adapted repertoire of carbohydrate-active enzymes for the utilization of pectin and related plant polysaccharides,^44,45^ and its enrichment may reflect increased utilization of broccoli-derived complex carbohydrates. Members of *Roseburia*, including *R. intestinalis*, are well-established butyrate-producing bacteria capable of utilizing plant-derived polysaccharides,^46,47^ and their higher abundance in the ND and FHB groups was consistent with the fecal butanoic acid profile. Consistent with this fiber-utilizing community, the broccoli matrix was rich in dietary fiber, comprising a soluble fraction of 5.2–5.3 g/100 g and a total dietary fiber content of 19.1–19.2 g/100 g (Supplementary Table S1).

Fecal short-chain fatty acid analysis showed that the HFFD group had significantly lower acetic acid, butanoic acid, and total SCFA concentrations than the ND group. Compared with the HFFD group, FHB supplementation significantly increased butanoic acid and total SCFA concentrations, whereas acetic acid showed an upward trend without a statistically significant difference (Figure 7G–I). Propanoic acid concentrations did not differ significantly among the HFFD-fed groups (Supplementary Figure S3). These results indicate a selective alteration in microbial fermentation products, more closely aligned with increased butyrate availability than with a generalized increase in all major SCFAs. Butyrate has been shown to influence skeletal muscle and whole-body metabolism by improving insulin sensitivity, stimulating mitochondrial oxidative metabolism, and acting as a histone deacetylase inhibitor.^48–50^ In aged mice, dietary butyrate also attenuated muscle loss and improved muscle-related metabolic characteristics.^49^ These mechanisms provide a biologically plausible basis for the coordinated occurrence of increased fecal butanoic acid and improved muscle-related phenotypes in the FHB group.

A limitation of the present study is that gut microbiota sequencing, fecal SCFA measurements, metabolic assessments, and skeletal muscle molecular analyses were conducted using different sets of animals, so individual-level correlations among differential microbial taxa, SCFA concentrations, and muscle-related phenotypes could not be evaluated. In addition, circulating SCFAs, direct butyrate intervention, fecal microbiota transplantation, antibiotic-mediated microbiota depletion, and gnotobiotic validation were not performed. Therefore, the observed microbiota remodeling, increased fecal butanoic acid, and altered skeletal muscle signaling should be interpreted as coordinated changes accompanying FHB supplementation rather than as evidence of a causal microbiota–SCFA–Akt/mTOR pathway.

In summary, *L. plantarum* fermentation altered the metabolite composition of broccoli, and the fermented product attenuated diet-induced adiposity, hepatic steatosis, and insulin resistance in HFFD-fed mice while improving muscle quality. These effects were accompanied by the enrichment of fermentation-derived metabolites and the remodeling of butyrate-producing gut microbiota. Further studies would help to clarify the specific components and mechanisms underlying these effects. These findings highlight the potential of fermentation to enhance the functional values of broccoli.

## Supplementary Material



## Data Availability

Data will be made available upon request.

## Notes

Table S1: Proximate composition of unfermented and fermented broccoli powders; Table S2: Composition and caloric distribution of the HFFD-based experimental diets; Table S3: Food intake, water intake, and calories; Table S4: Body weight and waistline; Table S5: Absolute and body-weight-normalized organ and tissue weights; Figure S1: Selection and in vitro metabolic-regulatory activity of *L. plantarum* SG066 in HepG2 cells; Figure S2: Physicochemical properties, phytochemical contents, and antioxidant capacity of the broccoli powders; Figure S3: Gut microbiota alpha-diversity indices and fecal propanoic acid concentration (PDF)

## Abbreviations

THβC-3-COOH: 2,3,4,9-tetrahydro-1*H*-β-carboline-3-carboxylic acid
4-HPLA: dl-4-hydroxyphenyllactic acid
ECL: enhanced chemiluminescence
FBG: fasting blood glucose
FHB: fermented high-dose broccoli
FLB: fermented low-dose broccoli
GRAS: generally recognized as safe
HDL-c: high-density lipoprotein cholesterol
HFFD: high-fat and high-fructose diet
HICA: 2-hydroxycaproic acid
H&E: hematoxylin and eosin
HRP: horseradish peroxidase
LDL-c: low-density lipoprotein cholesterol
PLA: l-(−)-3-phenyllactic acid
ND: normal diet
PVDF: polyvinylidene difluoride
SO: reactive oxygen species
SCFAs: short-chain fatty acids
TMB: tetramethylbenzidine
TC: total cholesterol
TFA: trifluoroacetic acid
TG: triglycerides
UHB.: unfermented high-dose broccoli
