# Supplementary material for: Lactiplantibacillus plantarum-Fermented Broccoli Attenuates Metabolic Dysfunction and Improves Skeletal Muscle Quality in High-Fat/High-Fructose Diet-Fed Mice
Source: J Agric Food Chem. 2026 Sep 28;74(39):31112–26. doi: 10.1021/acs.jafc.6c05322 (PMC13651633; doi:10.1021/acs.jafc.6c05322)
Supplement: Supplementary file 1 [file jf6c05322_si_001.pdf]

# Supporting Information

## *Lactiplantibacillus plantarum*-fermented broccoli attenuates metabolic dysfunction and improves skeletal muscle quality in high-fat/high-fructose diet-fed mice

Ya-Ru Kuo[1], Tzu-Ling Lin[1], Pin-Yu Ho[1], Wei-Jen Chen[2], Hui-Fang Chu[2], Ming-Chung Tseng[2], Yu-Shan Wei[2], Min-Hsiung Pan[1,3,4*]

**Table S1. Proximate composition of unfermented and fermented broccoli powders**

| Component (g/100 g) | Unfermented broccoli powder | Fermented broccoli powder |
|---|---|---|
| Moisture | 5.6 | 11 |
| Ash | 4.9 | 5.2 |
| Crude protein | 25.5 | 25.5 |
| Crude fat | 2.4 | 1.2 |
| Total dietary fiber | 19.2 | 19.1 |
| Soluble dietary fiber | 5.2 | 5.3 |
| Insoluble dietary fiber | 14 | 13.8 |

Moisture, ash, crude protein, crude fat, and total, soluble, and insoluble dietary fiber contents (g/100 g) of the unfermented and fermented broccoli powders. Moisture was determined by CNS 5033, ash by CNS 5034, crude protein by AOAC 992.15 (nitrogen × 6.25), crude fat by CNS 5036, and total, soluble, and insoluble dietary fiber by AOAC 991.43. Analyses were performed by SGS Taiwan Ltd. (Kaohsiung, Taiwan).

**Table S2. Composition and caloric distribution of the HFFD-based experimental diets**

| Diet group | D12451 (g/1000 g dry diet) | Broccoli powder (g/1000 g dry diet) | Protein (g/100 g) | Fat (g/100 g) | Carbohydrate (g/100 g) | Energy (kcal/100 g) | Protein (% kcal) | Fat (% kcal) | Carbohydrate (% kcal) |
|---|---|---|---|---|---|---|---|---|---|
| HFFD | 1000 | 0 | 23.7 | 23.6 | 40.3 | 468.4 | 20.2 | 45.4 | 34.4 |
| HFFD + 5% unfermented broccoli (UHB) | 950 | 50 | 23.8 | 22.5 | 41.4 | 463.5 | 20.5 | 43.8 | 35.7 |
| HFFD + 1% fermented broccoli (FLB) | 990 | 10 | 23.7 | 23.4 | 40.5 | 467.1 | 20.3 | 45.0 | 34.7 |
| HFFD + 5% fermented broccoli (FHB) | 950 | 50 | 23.8 | 22.5 | 41.1 | 462.0 | 20.6 | 43.8 | 35.6 |

The high-fat/high-fructose diet (HFFD; Research Diets D12451) was used alone or supplemented with 5% unfermented (UHB), 1% fermented (FLB), or 5% fermented (FHB) broccoli powder. Broccoli powders were incorporated at the expense of an equivalent weight of the D12451 base diet (50 g/1000 g for the 5% groups; 10 g/1000 g for the 1% group). Nutrient values were calculated from the proportional contributions of D12451 and the broccoli powders (Table S1). Carbohydrate contents were calculated by difference and included dietary fiber. Energy and caloric distributions were calculated using conversion factors of 4, 9, and 4 kcal/g for protein, fat, and carbohydrate, respectively. Water added during pellet preparation was removed by freeze-drying and excluded from the final dry-weight composition.

**Table S3. Effect of fermented broccoli on food intake, water intake and calories in HFFD-fed C57BL/6J mice**

| | ND | HFFD | UHB | FLB | FHB |
|---|---|---|---|---|---|
| **Food intake (g)** | 3.7 ± 0.6 $^{a}$ | 2.2 ± 0.5 $^{b}$ | 2.1 ± 0.3 $^{b}$ | 2.1 ± 0.4 $^{b}$ | 2.1 ± 0.3 $^{b}$ |
| **Calories of diet (kcal/g)** | 3.35 | 4.68 | 4.62 | 4.67 | 4.61 |
| **Water intake (mL)** | 5.1 ± 0.5 $^{abc}$ | 4.8 ± 0.5 $^{c}$ | 5.0 ± 0.5 $^{bc}$ | 5.5 ± 0.7 $^{ab}$ | 5.6 ± 0.9 $^{a}$ |
| **Calories of water (kcal/mL)** | - | 0.8 | 0.8 | 0.8 | 0.8 |
| **Food calories (kcal/day)** | 12.2 ± 1.9 $^{b}$ | 14.2 ± 2.4 $^{a}$ | 13.8 ± 1.6 $^{a}$ | 13.9 ± 2.1 $^{a}$ | 14.0 ± 1.7 $^{a}$ |
| **Energy efficiency ratio (%)** | 0.8 ± 0.1 $^{c}$ | 1.6 ± 0.1 $^{a}$ | 1.3 ± 0.1 $^{b}$ | 1.2 ± 0.3 $^{b}$ | 1.2 ± 0.2 $^{b}$ |

Energy efficiency ratio = body weight gain (g) / energy intake (kcal). Data are expressed as means ± SEM ($n$ = 8 per group). The significance of the difference was analyzed by one-way ANOVA followed by Duncan's multiple range test. Values with different letters (a–c) within a row are significantly different ($p < 0.05$) among groups.

**Table S4. Effects of fermented broccoli on body weight and waistline in HFFD-fed C57BL/6J mice**

| | ND | HFFD | UHB | FLB | FHB |
|---|---|---|---|---|---|
| **Initial weight (g)** | 19.2 ± 0.8 $^{a}$ | 20.1 ± 1.6 $^{a}$ | 19.5 ± 1.1 $^{a}$ | 19.8 ± 0.9 $^{a}$ | 19.3 ± 0.6 $^{a}$ |
| **Final weight (g)** | 28.5 ± 0.8 $^{c}$ | 42.9 ± 2.6 $^{a}$ | 37.5 ± 1.2 $^{b}$ | 37.1 ± 4.6 $^{b}$ | 36.2 ± 3.7 $^{b}$ |
| **Weight gain (g)** | 9.3 ± 1.1 $^{c}$ | 22.8 ± 2.1 $^{a}$ | 18.0 ± 1.6 $^{b}$ | 17.3 ± 3.8 $^{b}$ | 16.9 ± 3.4 $^{b}$ |
| **Waistline (cm)** | 5.4 ± 0.3 $^{c}$ | 6.4 ± 0.2 $^{a}$ | 6.1 ± 0.5 $^{ab}$ | 5.9 ± 0.4 $^{b}$ | 5.8 ± 0.3 $^{b}$ |

Data are expressed as means ± SEM ($n$ = 8 per group). The significance of the difference was analyzed by one-way ANOVA followed by Duncan's multiple range test. Values with different letters (a–c) within a row are significantly different ($p < 0.05$) among groups.

**Table S5. Effects of fermented broccoli on organ and tissue weights in HFFD-fed C57BL/6J mice**

| Organ / Tissue weight | ND | HFFD | UHB | FLB | FHB |
|---|---|---|---|---|---|
| **Liver (g)** | 1.38 ± 0.07 $^{c}$ | 1.93 ± 0.09 $^{a}$ | 1.64 ± 0.04 $^{b}$ | 1.63 ± 0.08 $^{b}$ | 1.52 ± 0.10 $^{bc}$ |
| **Liver/BW (mg/g)** | 48.20 ± 2.07 $^{a}$ | 45.49 ± 1.66 $^{a}$ | 44.75 ± 0.94 $^{a}$ | 44.11 ± 1.44 $^{a}$ | 43.72 ± 2.20 $^{a}$ |
| **Kidney (g)** | 0.38 ± 0.01 $^{b}$ | 0.41 ± 0.01 $^{a}$ | 0.37 ± 0.01 $^{b}$ | 0.38 ± 0.01 $^{b}$ | 0.36 ± 0.01 $^{b}$ |
| **Kidney/BW (mg/g)** | 13.02 ± 0.20 $^{a}$ | 9.85 ± 0.43 $^{c}$ | 9.91 ± 0.19 $^{c}$ | 10.94 ± 0.37 $^{b}$ | 10.06 ± 0.33 $^{bc}$ |
| **Spleen (g)** | 0.07 ± 0.00 $^{c}$ | 0.09 ± 0.00 $^{a}$ | 0.08 ± 0.00 $^{b}$ | 0.08 ± 0.00 $^{b}$ | 0.08 ± 0.00 $^{b}$ |
| **Spleen/BW (mg/g)** | 2.25 ± 0.09 $^{a}$ | 2.12 ± 0.09 $^{a}$ | 2.15 ± 0.06 $^{a}$ | 2.19 ± 0.08 $^{a}$ | 2.07 ± 0.08 $^{a}$ |
| **Perigonadal adipose tissue (g)** | 0.37 ± 0.03 $^{c}$ | 2.07 ± 0.07 $^{a}$ | 1.46 ± 0.22 $^{b}$ | 1.36 ± 0.23 $^{b}$ | 1.20 ± 0.14 $^{b}$ |
| **Perigonadal adipose tissue/BW (mg/g)** | 12.83 ± 0.99 $^{c}$ | 48.85 ± 1.34 $^{a}$ | 39.40 ± 5.52 $^{ab}$ | 36.16 ± 4.44 $^{b}$ | 33.92 ± 3.13 $^{b}$ |
| **Retroperitoneal adipose tissue (g)** | 0.11 ± 0.01 $^{c}$ | 1.02 ± 0.06 $^{a}$ | 0.67 ± 0.10 $^{b}$ | 0.56 ± 0.12 $^{b}$ | 0.55 ± 0.06 $^{b}$ |
| **Retroperitoneal adipose tissue/BW (mg/g)** | 3.70 ± 0.36 $^{c}$ | 23.80 ± 1.03 $^{a}$ | 18.05 ± 2.52 $^{b}$ | 14.59 ± 2.57 $^{b}$ | 15.62 ± 1.41 $^{b}$ |
| **Visceral adipose tissue (g)** | 0.48 ± 0.04 $^{c}$ | 3.09 ± 0.11 $^{a}$ | 2.13 ± 0.31 $^{b}$ | 1.92 ± 0.34 $^{b}$ | 1.75 ± 0.20 $^{b}$ |
| **Visceral adipose tissue/BW (mg/g)** | 16.54 ± 1.32 $^{c}$ | 72.64 ± 1.71 $^{a}$ | 57.45 ± 7.92 $^{b}$ | 50.75 ± 6.81 $^{b}$ | 49.55 ± 4.40 $^{b}$ |
| **Quadriceps (mg)** | 13.46 ± 0.77 $^{a}$ | 9.13 ± 0.42 $^{b}$ | 10.42 ± 1.03 $^{b}$ | 11.13 ± 0.84 $^{ab}$ | 11.24 ± 0.74 $^{ab}$ |
| **Quadriceps/BW (mg/g)** | 0.46 ± 0.02 $^{a}$ | 0.22 ± 0.01 $^{c}$ | 0.28 ± 0.03 $^{bc}$ | 0.32 ± 0.03 $^{b}$ | 0.33 ± 0.02 $^{b}$ |
| **Gastrocnemius (mg)** | 10.12 ± 0.59 $^{a}$ | 7.59 ± 0.21 $^{b}$ | 8.17 ± 0.28 $^{b}$ | 9.69 ± 0.56 $^{a}$ | 9.47 ± 0.35 $^{a}$ |
| **Gastrocnemius/BW (mg/g)** | 0.34 ± 0.02 $^{a}$ | 0.18 ± 0.01 $^{c}$ | 0.22 ± 0.01 $^{c}$ | 0.29 ± 0.02 $^{b}$ | 0.27 ± 0.01 $^{b}$ |
| **Soleus muscles (mg)** | 0.66 ± 0.05 $^{a}$ | 0.44 ± 0.06 $^{c}$ | 0.49 ± 0.04 $^{bc}$ | 0.54 ± 0.05 $^{abc}$ | 0.63 ± 0.03 $^{ab}$ |
| **Soleus muscles/BW (mg/g)** | 0.02 ± 0.00 $^{a}$ | 0.01 ± 0.00 $^{d}$ | 0.01 ± 0.00 $^{cd}$ | 0.02 ± 0.00 $^{bc}$ | 0.02 ± 0.00 $^{b}$ |

Data are expressed as means ± SEM ($n$ = 8 per group). The significance of the difference was analyzed by one-way ANOVA followed by Duncan's multiple range test. Values with different letters (a–d) within a row are significantly different ($p < 0.05$) among groups.

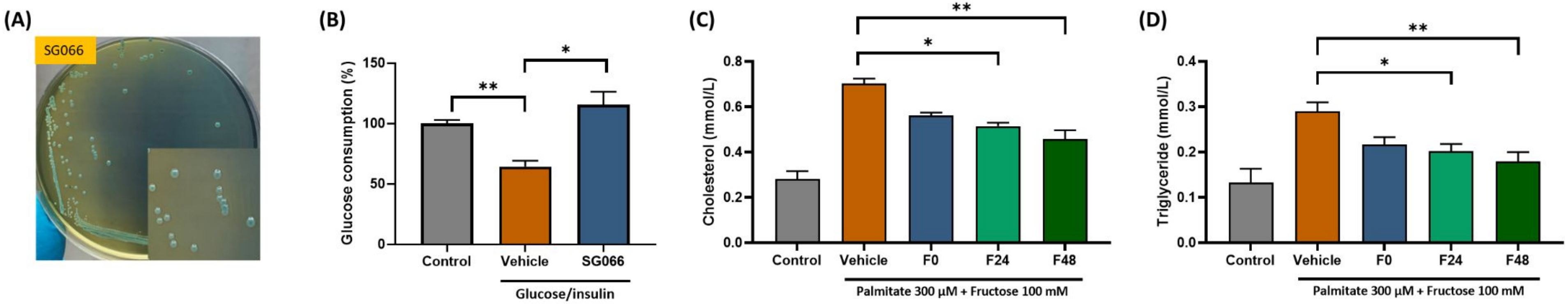


**Figure S1. Selection and *in vitro* metabolic-regulatory activity of *L. plantarum* SG066.** (A) Representative colony morphology of *L. plantarum* SG066 on agar. (B) Glucose consumption (%) in insulin-resistant HepG2 cells. HepG2 cells were rendered insulin-resistant with 35 mM glucose and 100 nM insulin and treated with vehicle or live SG066 ($1 \times 10^6$ CFU/mL) for 96 h; glucose consumption was normalized to cell viability (MTT). (C) Cholesterol and (D) triglyceride content in HepG2 cells treated with palmitate (300 μM) and fructose (100 mM), followed by SG066 fermentation products collected at 0 (F0), 24 (F24), and 48 (F48) h of fermentation. Control, untreated cells; Vehicle, palmitate/fructose challenge without treatment. Data are presented as mean ± SEM ($n = 3$ per group). Statistical significance was assessed by Student's *t*-test; *$p < 0.05$, **$p < 0.01$.

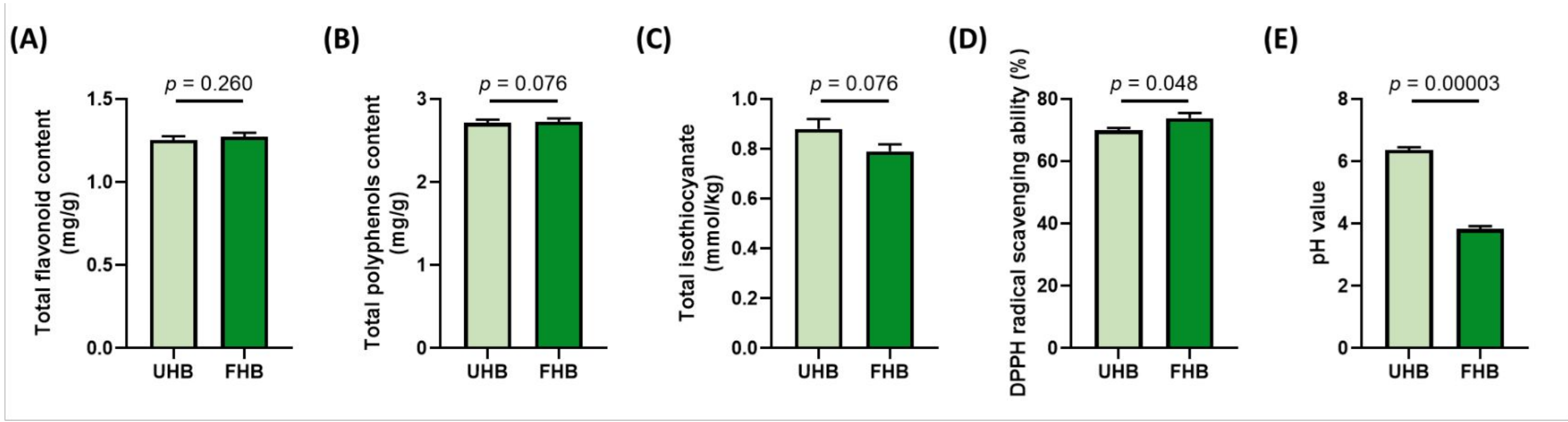


**Figure S2. Physicochemical properties, phytochemical contents, and antioxidant capacity of unfermented (UHB) and fermented (FHB) broccoli powders.** (A) Total flavonoid content (mg/g), (B) total polyphenol content (mg/g), (C) total isothiocyanate content (mmol/kg), (D) DPPH radical scavenging ability (%), and (E) pH value were measured in unfermented and fermented broccoli powders. Data are presented as mean ± SEM (n = 3 per group). Statistical significance was assessed by Student's t-test; p values are indicated above each comparison.

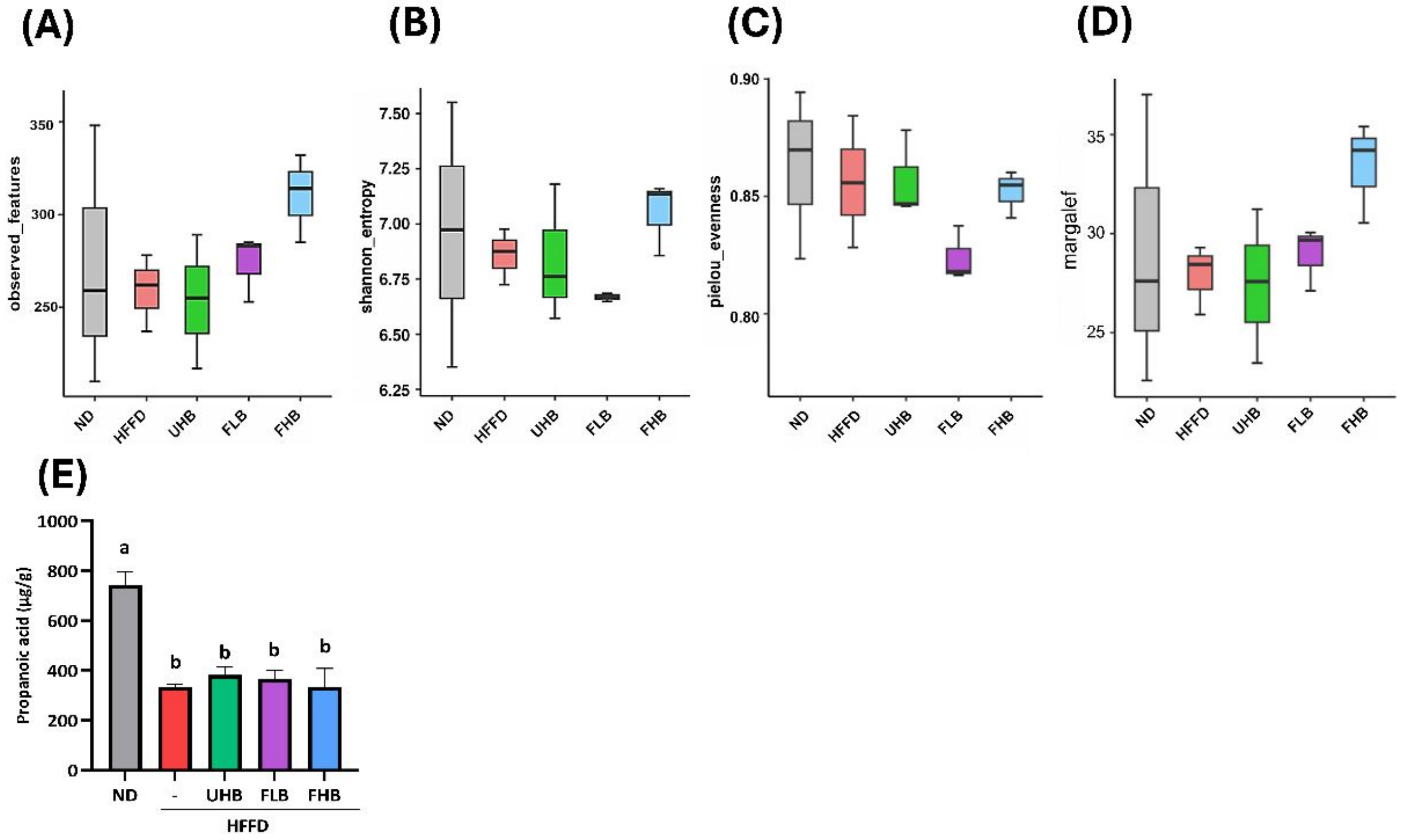


**Figure S3. Fermented broccoli modulates the composition of the gut microbiota and production of short-chain fatty acids (SCFAs) in HFFD-fed C57BL/6J mice.** Alpha-diversity indices of the gut microbiota and fecal propanoic acid concentration in HFFD-fed C57BL/6J mice. (A–D) Alpha-diversity indices of the gut microbiota, including (A) observed features, (B) Shannon entropy, (C) Pielou's evenness, and (D) Margalef's richness index. (E) Fecal propanoic acid concentration. Data for the diversity indices are presented as box plots (median with interquartile range), and the propanoic acid concentration is expressed as mean ± SEM (*n* = 3 per group).
